# Estimation of General Practitioner Visits, Hospitalizations and Deaths Attributable to Respiratory Syncytial Virus and Influenza Virus, and Costs Associated With Hospitalizations, in Older Adults in France From 2010 to 2020

**DOI:** 10.1093/ofid/ofaf735

**Published:** 2025-12-05

**Authors:** Charles Nuttens, Vanessa Barbet, Clélia Bignon-Favary, Emilie Lambourg, Stéphane Fiévez, Emmanuelle Blanc, Mathias Vacheret, Hervé Liliu, Philippe Vanhems, Jean-Sébastien Casalegno, Laurence Watier, Paul Loubet, Caihua Liang, Elizabeth Begier, Magali Lemaitre

**Affiliations:** Medical Affairs Vaccines, Pfizer, Paris, France; Horiana, Bordeaux, France; Horiana, Bordeaux, France; Horiana, Bordeaux, France; Medical Affairs Vaccines, Pfizer, Paris, France; Medical Affairs Vaccines, Pfizer, Paris, France; Medical Affairs Vaccines, Pfizer, Paris, France; Inbeeo, London, UK; Service Hygiène, Epidémiologie et Prévention, Hospices Civils de Lyon, Lyon, France; Centre International de Recherche en Infectiologie, Univ Lyon, Inserm, U1111, Université Claude Bernard Lyon, CNRS, UMR5308, ENS de Lyon, Lyon, France; Hospices Civils de Lyon, Hôpital de la Croix-Rousse, Centre de Biologie Nord, Institut des Agents Infectieux, Laboratoire de Virologie, Lyon, France; Epidemiology and Modelling of Bacterial Escape to Antimicrobials, Institut Pasteur, Paris, France; VBIC, INSERM U1047, Univ Montpellier, Service des Maladies Infectieuses et Tropicales, CHU Nîmes, Nîmes, France; Vaccines Evidence Generation, Pfizer, Collegeville, Pennsylvania, USA; Medical Affairs Vaccines, Pfizer, Dublin, Ireland; Horiana, Bordeaux, France; Health Data Expertise, Génissieux, France

**Keywords:** France, incidence, influenza, RSV, time-series modeling

## Abstract

**Background:**

Respiratory syncytial virus (RSV) and influenza virus are leading causes of respiratory infections, causing substantial numbers of hospitalizations and deaths, particularly among vulnerable groups such as infants and older adults. While the burden of influenza virus infection is well-documented, the burden of RSV infection in the adult population is often underestimated due to diagnostic challenges and infrequent standard-of-care testing. This study aimed to estimate the incidence of general practitioner (GP) visits, hospitalizations, and deaths attributable to RSV and influenza virus infections in French adults aged ≥50 years, with a focus on ≥65 years, using a time-series model-based approach. In addition, costs associated with hospitalizations were calculated.

**Methods:**

Cyclic Poisson regression models and weekly data from French medical administrative databases and electronic medical records over ten epidemic seasons (2010–2020) were used to estimate incidences for RSV and influenza. The results were stratified by age group, diagnosis causes (respiratory and cardiorespiratory) and diagnosis type (primary and secondary). Average costs per hospitalization were calculated and multiplied by the number of hospitalizations estimated.

**Results:**

Among adults aged ≥65 years, we estimated RSV infection was responsible for 647 619 GP visits 24 319 hospitalizations, and 878 deaths per year. Incidence rates for GP visits for RSV were twice as large as for influenza; hospitalization rates were similar and mortality was lower. The mean annual cost of RSV-attributable hospitalizations was 105 million €, similar to influenza.

**Conclusions:**

This study highlighted the burden of RSV disease in the adult population in France is higher than previous reported. We envisage that this model-based approach will be instrumental in evaluating the impact of RSV vaccination campaigns.

## Graphical Abstract

**Graphical Abstract:**
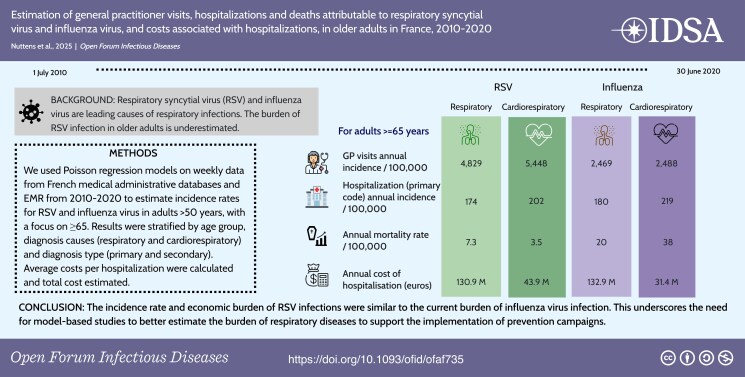
This graphical abstract is also available at Tidbit: https://tidbitapp.io/tidbits/estimation-of-general-practitioner-visits-hospitalizations-and-deaths-attributable-to-respiratory-syncytial-virus-and-influenza-and-costs-associated-with-hospitalizations-in-older-adults-in-france-2010-2020?utm_campaign=tidbitlinkshare&amp;utm_source=IO.

Respiratory syncytial virus (RSV) and influenza virus infections typically cause mild illness, with flu-like symptoms such as coughing, rhinorrhoea, and wheezing. However, they can also lead to severe respiratory complications, decompensation of underlying diseases, and even death in vulnerable populations like infants, older adults, immunocompromised individuals, and patients with comorbidities [1–4]. While the burden of influenza has been extensively documented in adults in France, the burden of adult RSV infection remains under-recognized [3, 5, 6].

Model-based approaches commonly used to study influenza epidemiology are increasingly applied in RSV research [7, 8]. These models use time-series analysis comparing weekly variations in hospitalizations or deaths and viral activity to estimate the proportion of events attributable to specific viruses [4, 6, 9]. In France, influenza-attributable respiratory hospitalizations in adults aged ≥65 years have been estimated with incidence rates ranging from 59 to 344 per 100 000 depending on the season [6]. For RSV, a recent systematic literature review, meta-analysis, and modeling study estimated a pooled annual incidence of RSV-related hospitalization in adults aged ≥65 years in high-income countries at 157 per 100 000 persons, with a corrected estimate of 347 per 100 000 when accounting for diagnostic limitations (eg, low sensitivity of single specimen testing, and differences in test type such as DFA vs PCR) [10]. A recent analysis of the French national hospital using RSV-specific ICD-10 codes in adults aged ≥18 years reported an incidence rate of 7.2 RSV hospitalizations per 100 000 adults annually between 2012 and 2021 [12]. This incidence rate is likely underestimated due to the non-specific symptoms of RSV infection, infrequent standard-of-care testing, low diagnostic accuracy, and misuse of RSV-specific ICD-10 codes [10–17]. In addition, adults hospitalized with RSV and influenza virus infections have elevated risks of cardiac events, with nearly one-quarter of hospitalized adults aged ≥50 years [18] and 11.7% of adults hospitalized with influenza [19] experiencing an acute cardiovascular event.

Our primary objective was to estimate the incidence of general practitioner (GP) visits, hospitalizations, and deaths for respiratory and cardiorespiratory cause attributable to RSV in adults aged ≥50 using multiple time-series regression modeling. We compared RSV estimates to influenza, examined incidence rates by age group with a focus on ≥65 years, characterized hospitalizations and calculated related costs.

## METHODS

### Study Design

This is a model-based study using general population data to estimate the annual number of GP visits, hospitalizations, and deaths attributable to RSV and influenza virus, and the rates per 100 000 population among French adults aged ≥65 years and for the age groups ≥50–64, 65–74, and ≥75 years. Costs of RSV- and influenza-associated hospitalizations were calculated by the average cost of hospitalization for each incidence rate estimate.

### Data Sources

#### General Practitioner Visits

**Figure 1.**
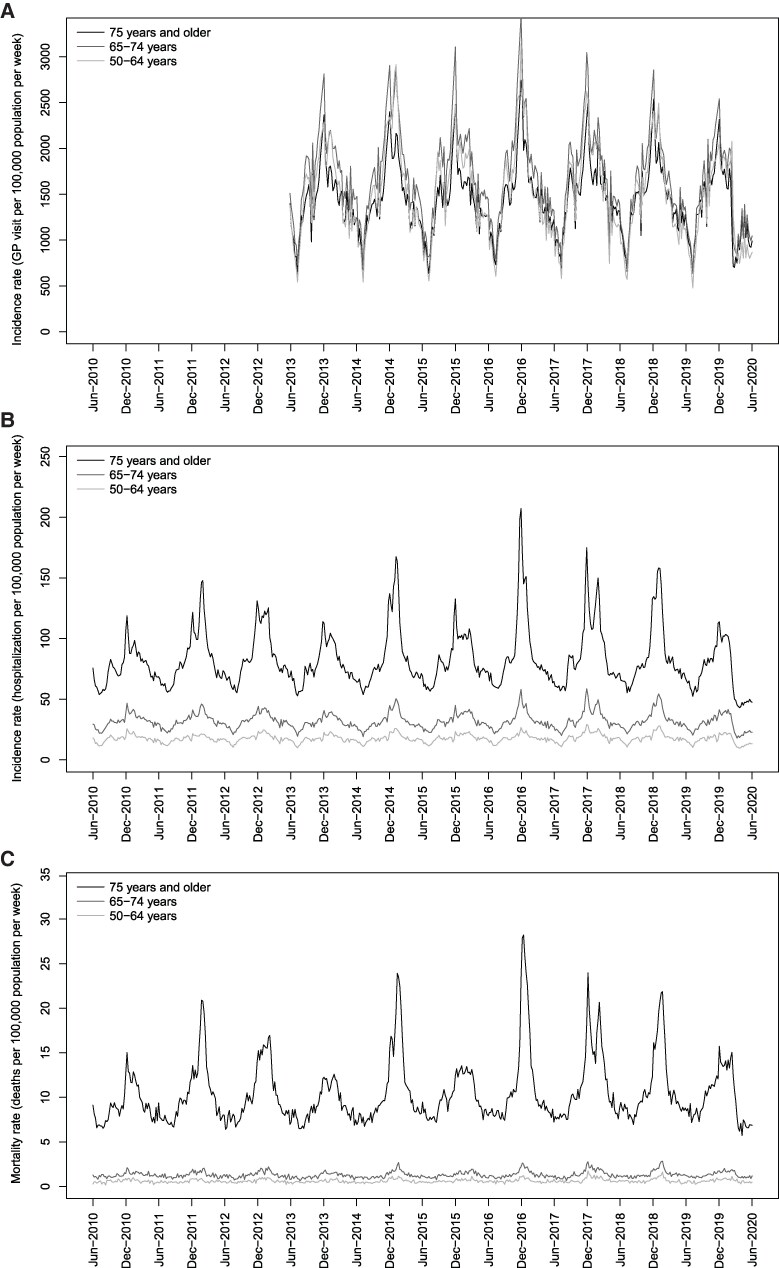
Weekly time-series of general practitioner visits, hospitalizations, and deaths by age group for respiratory causes (2010–2020). Evolution of the weekly number of general practitioner (GP) visits (*A*), hospitalizations (*B*), and deaths (*C*) per 100 000 population for respiratory causes (ICD-10 codes: J00–J99) as the primary diagnosis in France between June 2013 and June 2020 (*A*) or between June 2010 and June 2020 (*B* and *C*) for individuals aged 50–64, 65–74, or ≥75 y.

Weekly numbers of GP visits for respiratory (ICD-10 codes: J00-J99) and cardiorespiratory (ICD-10 codes: I21, I50, I63, I64, J00–J99) causes, stratified by age group, were obtained from the GP network Electronic Medical Records (EMR) database (Figure 1, Supplementary Figure 1). Cardiac codes were selected based on their associations with cardiac manifestations of RSV and influenza virus infection (Supplementary Table 1) [18–20]. The EMR database collects EMR from 1200 GPs in France (representing approximately 2% of all French GPs). The panel of contributing GPs is representative of the primary care physician population, based on three main criteria: age, sex, and geographical region.

#### Hospitalizations

Weekly numbers of hospitalizations for respiratory (ICD-10 codes: J00–J99), cardiorespiratory (ICD-10 codes: I00–I99, J00–J99), and narrowly-defined cardiorespiratory (ICD-10 codes: I21, I50, I63, I64, J00–J99) causes, stratified by age group, were extracted from the French national hospital database (Programme de Médicalisation des Systèmes d’Information [PMSI]) (Figure 1, Supplementary Figure 1, Supplementary Table 1). Hospitalizations were identified using the primary diagnosis code alone (ie, code used to describe the main reason for admission) and primary and secondary diagnoses codes (ie, codes used to report complications during hospitalization, laboratory test results, or any comorbidities). For the characterization and economic burden estimation of RSV hospitalizations, we included adults aged ≥50 years with at least one primary or secondary RSV- (ie, J121, J205, J210, B974), or influenza-related (ie, J09, J10, J11) ICD-10 code (Supplementary Table 1).

#### Deaths

Weekly numbers of deaths from respiratory (ICD-10 codes: J00–J99) or cardiorespiratory (ICD-10 codes: I00–I99, J00–J99) causes were extracted from the National Epidemiology Centre of Medical Causes of Death (CépiDc) database, which includes death certificates for the entire French population [21].

### Study Period

Data on the weekly numbers of hospitalizations and deaths were extracted from the PMSI and the CépiDc databases from the first week of July 2010 to the last week of June 2020 (spanning 10 epidemic seasons). Data on the weekly number of GP visits were collected from the first week of July 2013 to the last week of June 2020 from the EMR database due to data access restrictions. For modeling, the last week of February 2020 was set as data cut-off date to exclude the COVID-19 pandemic period.

### Indicators of Respiratory Syncytial Virus and Influenza Virus Activity

**Figure 2.**
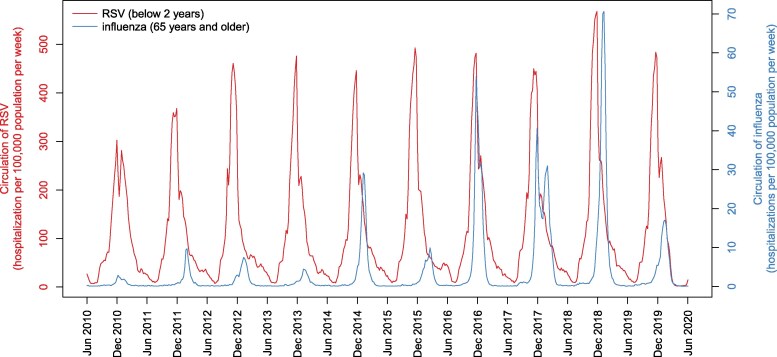
Weekly time-series of respiratory syncytial virus and influenza virus circulation (2010–2020). Evolution of the weekly number of hospitalizations per 100 000 population due to respiratory syncytial virus (RSV) infection among children aged ≤2 y (ICD-10 codes: J205, J210, J219) or influenza virus infection among adults aged ≥65 y (ICD-10 codes: J09, J10, J11) in France between June 2010 and June 2020.

Weekly numbers of hospitalizations due to RSV (ICD-10 codes: J20.5, J21.0, J21.9) among children aged <2 years and due to influenza virus (ICD-10 codes: J09, J10, J11) among individuals aged ≥65 years were extracted from the PMSI database as indicators of RSV and influenza virus circulation, respectively (Figure 2 and Supplementary Table 1).

Further information on the model, time lags (Supplementary Table 2), model fit (Supplementary Table 3), incidence estimation, and data sourcing can be found in the Supplementary material.

### Estimation of Hospitalization Costs

Average annual hospitalization costs were calculated from public health expenditure, using the PMSI database. Economic burdens attributable to RSV and influenza were estimated, for the different age groups, by multiplying the mean hospital cost per RSV- or influenza-associated hospital stay by the estimated incidence of hospitalization using the INSEE French population data. As RSV hospitalization costs varied significantly across seasons due to testing and coding changes, the final analysis used data from the last two RSV seasons (2017–2019), considered more representative of actual costs.

### Statistical Analysis

Age- and cause-specific hospitalization data were analyzed using a model that incorporated indicators of RSV/influenza activity, time trends, and the seasonal term variations. The model employed the following log link function where is the weekly number of hospitalization or deaths from cause “*c*” in age group “*a*”. Terms *t*, *t*², and *t*^3^ are the linear, quadratic, and cubic terms of the time trend, respectively:


$$\begin{array}{rl} log(H_{t}^{\left(c,a\right)})\;= & \;\alpha_{0}+\alpha_{1}t+\alpha_{2}t^{2}+\alpha_{3}t^{3}+\;\overset{12}{\underset{i=1}{\sum }}\;[\beta_{i}cos\left(\frac{it\pi }{52,14}\right)\\ & +\gamma_{i}sin\left(\frac{it\pi }{52,14}\right)]+\;\eta lweek\\ & +\overset{10}{\underset{i=1}{\sum }}\;\delta_{i}ILI_{t-l}+\theta RSV_{t-l} \end{array}$$

where $H_{t}^{\left(c,a\right)}$ is the weekly number of events per 100 000 persons for cause ***c***, in a specific age group ***a***, during the week ***t***. Terms *t*, *t*², and *t*^3^ are the linear, quadratic, and cubic terms of the time trend, respectively. The terms cos and sin represent seasonality. A binary variable **lweek**, which represents the last week of the year (as it includes two bank holidays: Christmas Day and New Year's Day) was integrated into the model. $RSV_{t}$ and $ILI_{t}$ represent the weekly indicator of RSV and influenza virus activity, respectively. Parameters *α*, *β*, *γ*, *δ*, and *θ* were estimated using a likelihood method.

All statistical analyses were performed using SAS, version 9.4 (SAS Institute Inc., Cary, NC, USA) and R, version 4.2.0 (The R Foundation, Vienna, Austria). We estimated the number of GP visits, hospitalizations, and deaths attributable to RSV and influenza virus infection using cyclic Poisson regression models which enabled us to estimate incidence and mortality rates using population data from INSEE (French national institute of statistics and economics studies). Weekly numbers of GP visits, hospitalizations, and deaths were modeled using a log link function to account for the baseline incidence of each outcome, time trends, seasonal terms, and independent indicators of RSV and influenza virus activity. Age- and cause-specific hospitalization data were interpreted using RSV/influenza virus circulation activity, time trends, and seasonal term.

## RESULTS

### General Practitioner Visit Incidence

Over 2013–2020, the estimated average annual incidence rate of GP visits attributable to RSV in adults aged ≥65 years was 4829/100 000 for respiratory causes and 5448/100 000 for cardiorespiratory causes (Table 1). This corresponded to 573 998 and 647 619 GP visits, representing 5.84% and 5.50% of total annual GP visits for this age group for respiratory and cardiorespiratory causes, respectively (Supplementary Table 4). These figures rose to 10.81% and 10.44% when restricting the timeframe to November to March each year (Northern Hemisphere winter). The incidence rate for respiratory-cause GP visits varied from 2736/100 000 among adults aged 50–64 years to 5396/100 000 among adults aged 65–75 years (Table 2, Supplementary Figure 3). The mean annual incidence of influenza-attributable GP visits was lower among adults aged ≥65 years (2469/100 000 for respiratory causes and 2488/100 000 for cardiorespiratory causes).

**Table 1.** Mean Estimated Incidence of General Practitioner Visits, Hospitalizations, and Deaths Attributable to Respiratory Syncytial Virus and Influenza Virus Infections, Using Respiratory or Cardiorespiratory Primary Diagnosis ICD-10 Codes

		RSV		Influenza Virus	
	Age group (y)	Number of cases [95% CI]	Incidence rate/100 000 population [95% CI]	Number of cases [95% CI]	Incidence rate/100 000 population [95% CI]
**GP VISITS (12-m average for 2013–2020)**					
**Respiratory disease** **(J00−J99)**	**50–64**	340 030 [335 100–345 152]	2736 [2696–2777]	479 748 [476 307–483 087]	3861 [3833–3888]
	**65–74**	343 795 [341 377–346 209]	5395 [5358–5433]	186 875 [185 007–188 720]	2958 [2928–2987]
	**≥75**	230 204 [228 313–232 297]	4179 [4144–4217]	105 048 [103 529–106 535]	1911 [1883–1938]
	**≥65^a^**	573 998 [569 690–578 505]	4829 [4792–4867]	291 923 [288 536–295 255]	2469 [2440–2497]
**Cardiorespiratory disease** **(I21, I50, I63, I64, J00−J99)**	**50–64**	946 591 [937 015–955 224]	7616 [7539–7685]	523 192 [519 282–526 891]	4211 [4180–4241]
	**65–74**	379 935 [377 436–382 475]	5962 [5923–6002]	203 994 [202 045–205 903]	3230 [3200–3260]
	**≥75**	267 685 [265 513–269 932]	4859 [4820–4900]	89 964 [88 245–91 669]	1638 [1606–1669]
	**≥65^a^**	647 619 [642 949–652 408]	5448 [5409–5489]	293 958 [290 290–297 573]	2488 [2457–2519]
**HOSPITALIZATIONS (12-m average for 2010–2020)**					
**Respiratory disease** **(J00−J99)**	**50–64**	4199 [3746–4656]	34 [30–38]	5482 [5184–5749]	44 [42–46]
	**65–74**	5696 [5467–5913]	93 [90–97]	5748 [5605–5880]	94 [92–96]
	**≥75**	15 208 [14 952–15 422]	256 [252–259]	15 985 [15 810–16 167]	268 [265–271]
	**≥65^a^**	20 904 [20 418–21 335]	174 [170–177]	21 732 [21 416–22 047]	180 [177–182]
**Narrowly-defined cardiorespiratory disease** **(I21, I50, I63, I64, J00−J99)**	**50–64**	5955 [5425–6461]	48 [44–52]	6001 [5658–6313]	48 [46–51]
	**65–74**	8007 [7728–8280]	131 [127–136]	6409 [6226–6583]	105 [102–108]
	**≥75**	16 312 [16 014–16 602]	274 [269–279]	20 072 [19 790–20 350]	337 [333–342]
	**≥65^a^**	24 319 [23 742–24 883]	202 [197–207]	26 481 [26 016–26 933]	219 [215–223]
**Cardiorespiratory disease** **(I00−I99, J00−J99)**	**50–64**	5346 [4072–6671]	43 [33–54]	4512 [3766–5174]	36 [30–42]
	**65–74**	7023 [6251–7747]	115 [103–127]	4945 [4512–5345]	82 [74–88]
	**≥75**	13 146 [12 720–13 548]	221 [214–228]	21 295 [20 920–21 628]	358 [352–364]
	**≥65^a^**	20 169 [18 971–21 295]	168 [158–177]	26 239 [25 432–26 973]	218 [211–224]
**DEATHS (12-m average for 2010–2020)**					
**Respiratory disease** **(J00−J99)**	**50–64**	11 [0–130]	0.1 [0.0–1.0]	191 [109–280]	1.5 [0.9–2.3]
	**65–74**	22 [0–108]	0.4 [0.0–1.8]	297 [229–360]	4.8 [3.7–5.8]
	**≥75**	856 [709–1011]	14 [12–17]	2104 [2027–2186]	35 [34–37]
	**≥65^a^**	878 [709–1120]	7.3 [5.8–9.3]	2401 [2256–2545]	20 [19–21]
**Cardiorespiratory disease** **(I00−I99, J00−J99)**	**50–64**	214 [93–332]	1.7 [0.8–2.7]	343 [249–441]	2.8 [2.0–3.6]
	**65–74**	427 [357–499]	7.0 [5.9–8.2]	499 [435–567]	8.2 [7.1–9.4]
	**≥75**	0 [0–129]	0.0 [0.0–2.2]	4051 [3950–4159]	68 [66–70]
	**≥65^a^**	427 [357–628]	3.5 [3.1–5.3]	4551 [4385–4726]	38 [36–39]

Abbreviations: CI, confidence interval; GP, general practitioner; RSV, respiratory syncytial virus.

^a^The incidence rates for the ≥65-y age group were estimated independently; thus, the number of cases do not correspond to the sum of the rates for the groups aged 65–74 and ≥75 y. Population estimates to transform number of cases to incidence rates per 100 000 population are from INSEE (French national institute for statistics and economics studies).

**Table 2.** Average Estimated Incidence of Hospitalizations Attributable to Respiratory Syncytial Virus and Influenza Virus Infections, Using Respiratory or Cardiorespiratory Primary and Secondary Diagnoses ICD-10 Codes

		RSV		Influenza Virus	
	Age group (y)	Number of cases [95% CI]	Incidence rate/100 000 population [95% CI]	Number of cases [95% CI]	Incidence rate/100 000 population [95% CI]
**HOSPITALIZATIONS (12-MONTH average for 2010–2020)**					
**Respiratory disease** **(J00−J99)**	**50–64**	700 [183–1212]	5.6 [1.5–9.8]	5662 [5157–6119]	46 [42–49]
	**65–74**	9255 [8770–9748]	152 [144–160]	6489 [6178–6761]	107 [101–111]
	**≥75**	17 256 [16 924–17 562]	290 [285–296]	22 365 [22 060–22 653]	376 [371–381]
	**≥65^a^**	26 511 [25 694–27 309]	220 [214–227]	28 855 [28 238–29 414]	239 [234–244]
**Narrowly-defined cardiorespiratory disease (I21, I50, I63, I64, J00−J99)**	**50–64**	8437 [7608–9267]	68 [61–75]	6290 [5738–6766]	51 [46–55]
	**65–74**	12 015 [11 491–12 543]	197 [189–206]	7586 [7253–7883]	125 [119–130]
	**≥75**	21 926 [21 519–22 298]	369 [362–375]	29 045 [28 693–29 370]	488 [482–494]
	**≥65^a^**	33 941 [33 010–34 841]	282 [275–290]	36 631 [35 946–37 252]	304 [298–309]
**Cardiorespiratory disease (I00–I99, J00−J99)**	**50–64**	6956 [4850–9119]	56 [39–74]	3059 [2227–4033]	25 [18–33]
	**65–74**	12 713 [11 327–14 183]	210 [187–234]	3283 [2637–3843]	56 [45–65]
	**≥75**	0 [0–0]	0.0 [0.0–0.0]	18 139 [17 519–18 669]	305 [295–314]
	**≥65^a^**	12 713 [11 327–14 183]	106 [94–118]	21 422 [20 156–22 511]	178 [168–188]

Abbreviations: CI, confidence interval; RSV, respiratory syncytial virus.

^a^The incidence rates for the ≥65-y age group were estimated independently; thus, the number of cases did not correspond to the sum of the rates for the groups aged 65–74 and ≥75 y. Population estimates to transform number of cases to incidence rates per 100 000 population are from INSEE (French national institute for statistics and economics studies).

### Hospitalization Incidence

Between 2010 and 2020, in adults aged ≥65 years, the mean annual hospitalization incidence rate attributable to RSV as primary diagnosis code was 174/100 000 for respiratory causes, 202/100 000 for narrowly-defined cardiorespiratory causes, and 168/100 000 for cardiorespiratory causes (Table 1). This corresponded to 6.00%, 3.26%, and 1.56% of annual hospitalizations for these causes, respectively, and 11.37%, 6.57%, and 3.23% when restricting the analysis to November-March (Supplementary Table 4). Hospitalization rates due to respiratory causes increased with age (50–64 years: 34/100 000; 65–74 years: 93/100 000; ≥ 75 years: 256/100 000) (Table 1, Supplementary Figure 3). Average annual hospitalizations for narrowly-defined cardiorespiratory disease for adults aged ≥65 years was 24 319 [23 742–24 883] (Table 1). Mean annual hospitalization incidence rates for influenza and RSV were comparable (Table 1).

When including secondary diagnosis codes, we found that 17.7% of patients hospitalized with RSV as a secondary diagnosis had no respiratory or cardiac codes as primary diagnosis (Supplementary Table 5) and were not captured in the primary code analysis. When both primary and secondary diagnosis codes were considered, the annual mean incidence rate of RSV-attributable hospitalization in adults aged ≥65 years was 220/100 000 for respiratory causes and 282/100 000 for narrowly-defined cardiorespiratory causes (Table 2), representing a 26% and 40% increase, respectively, compared with primary diagnosis codes alone.

### Mortality

Over 2010–2020, the estimated mean annual mortality rate attributable to RSV in adults aged ≥65 years was 7.3/100 000 for respiratory causes and 3.5/100 000 for cardiorespiratory causes, corresponding to 878 and 427 deaths per year (Table 1). The mean annual mortality rate due to RSV-associated respiratory causes increased with age (50–64 years: 0.1 deaths per 100 000; 65–74 years: 0.4/100 000; ≥ 75 years: 14/100 000) (Table 1, Supplementary Figure 3). Annual mortality rates were higher for cardiorespiratory causes than for respiratory causes in the 50–64 and 65–74 age groups (Table 1). In individuals aged ≥75 years, RSV mortality estimates for cardiorespiratory causes were not generated due to poor model fit, impacting the estimates among adults ≥65 years. Overall, 2.47% of the annual, and 4.52% of seasonal deaths for respiratory causes were attributable to RSV (Supplementary Table 4). The estimated average annual mortality attributable to influenza in adults aged ≥65 years was higher than that attributable to RSV (20/100 000 for respiratory causes and 38/100 000 for cardiorespiratory causes) (Table 1).

Observed and predicted weekly rates for GP visits, hospitalizations and mortality for respiratory and cardiorespiratory causes across age-groups are shown in Supplementary Figure 4a–f.

### Hospitalization Characteristics

Directly examining RSV and influenza hospitalizations from 2015 to 2019 diagnosed by standard-of-care testing, we identified 10 048 RSV-related and 108 657 influenza-related hospitalizations among adults aged ≥50 years (Table 3). For adults aged ≥65 years, there were 8183 and 87 867 hospitalizations coded for RSV and influenza virus respectively (Table 3). Annual RSV hospitalizations for adults aged ≥65 years were underestimated by a factor of around 12 compared with the modeling approach. Overall, 60.8% of RSV and 60.2% of influenza hospitalizations involved patients aged ≥75 years (Table 3).

**Table 3.** Characterization of Hospitalizations With Respiratory Syncytial Virus Diagnoses (ICD-10 Codes: J12.1, J20.5, J21.0, B974) or Influenza Virus Diagnoses (ICD-10 Codes: J09, J10, J11) Infection, by Age Group (2015–2019)

	RSV				Influenza Virus			
	Aged 50–64 y (N = 1865)	Aged 65–74 y (N = 2071)	Aged ≥75 y (N = 6112)	Aged ≥65 y (N = 8183)	Aged 50–64 y (N = 20 790)	Aged 65–74 y (N = 22 381)	Aged ≥75 y (N = 65 486)	Aged ≥65 y (N = 87 867)
**Duration of hospitalization (d)**								
Mean (SD)	12.3 (15.2)	12.5 (14.2)	12.0 (10.4)	12.1 (11.5)	10.0 (14.8)	10.6 (15.2)	10.8 (10.2)	10.7 (11.7)
Median (Q1—Q3)	8.0 (4.0–14.0)	8.0 (5.0–15.0)	9.0 (6.0–15.0)	9.0 (5.0–15.0)	6.0 (2.0–11.0)	7.0 (3.0–13.0)	8.0 (4.0–14.0)	8.0 (4.0–14.0)
Min–Max	0–192	0–145	0–124	0–145	0–305	0–898	0–186	0–898
**Disease severity, N (%)**								
At least one stay in intensive care, continuous care, or resuscitation	696 (37.3)	742 (35.8)	1162 (19.0)	1904 (23.3)	6223 (29.9)	5945 (26.6)	8789 (13.4)	14 734 (16.8)
In-hospital deaths	93 (5.0)	130 (6.3)	558 (9.1)	688 (8.4)	933 (4.5)	1332 (6.0)	5572 (8.5)	6904 (7.9)
**3-m readmission, N (%)**								
All causes	931 (52.5)	968 (49.9)	2140 (38.5)	3108 (41.5)	7061 (35.6)	8123 (38.6)	19 329 (32.3)	27 452 (33.9)
Cardiorespiratory causes	291 (16.4)	316 (16.3)	862 (15.5)	1178 (15.7)	1488 (7.5)	1983 (9.4)	5976 (10.0)	7959 (9.8)
Respiratory causes	261 (14.7)	263 (13.5)	588 (10.6)	851 (11.4)	1247 (6.3)	1514 (7.2)	3794 (6.3)	5308 (6.6)

Abbreviations: IQR, interquartile range; RSV, respiratory syncytial virus; SD, standard deviation.

In patients aged ≥65 years, the average length of hospital stay was 12.1 days for RSV and 10.7 days for influenza. The proportion of patients hospitalized for RSV requiring intensive care, continuous care, or resuscitation units decreased with age (50–64 years: 37.3%; 65–74 years: 35.8%; ≥ 75 years: 19.0%) (Table 3). A similar trend was observed for influenza hospitalizations with lower rates. In-hospital mortality increased with age for both RSV (50–64 years: 5.0%; ≥ 75 years: 9.1%), and influenza (50–64 years: 4.5%; ≥ 75 years: 8.5%).

### Economic Burden of Respiratory Syncytial Virus and Influenza-attributable Hospitalizations

Among patients aged ≥50 years, the average annual cost of hospitalization attributable to RSV infection was 130.9 million € for hospitalizations with a respiratory cause and 43.9 million € for hospitalizations with a cardiac cause, both as the main diagnosis (Supplementary Table 6). Patients aged ≥65 years accounted respectively for 80.1% and 59.5% of the total costs. Among patients aged ≥50 years, the average annual cost of hospitalization attributable to influenza virus infection was 132.9 million € for hospitalizations with a respiratory cause and 31.4 million € for hospitalizations with a cardiac cause, both as the main diagnosis. Hospitalization cost estimates for influenza were similar to those for RSV (Supplementary Table 6).

## DISCUSSION

We estimated the incidence of RSV-attributable GP visits, hospitalizations and mortality, among adults aged ≥50 years in France. Previous hospitalization estimates based on RSV-specific ICD-10 codes have underestimated RSV burden due to limited testing, low diagnostic accuracy, and misclassification [22, 23]. Using our model-based approach, we estimated ∼25 000 RSV hospitalizations annually among adults ≥65 years —about 13 times higher than what ICD-10 coding alone would suggest (∼2000 cases/year). RSV hospitalization incidence among adults ≥65 years ranged from 174 to 282/100 000. Higher rates were reported in the U.K., Spain, and Germany (234–787/100 000) [7, 8, 13]. A pooled analysis of U.S. studies reported an adjusted RSV hospitalization incidence rate for this population of 282/100 000 for prospective studies and 235.7/100 000 for time-series analyses [24] after adjustment for diagnostic testing-based under-ascertainment. Another meta-analysis reported a lower pooled rate of 150/100 000 among individuals aged ≥65 years [5], though this result was influenced by the healthy volunteer effect of clinical trials and methodological variations (primary and secondary diagnoses, inclusion of respiratory and cardiorespiratory hospitalization causes). In our analyses, we examined the impact of parameters on the model, and incidence rates. Standardizing methodologies across countries could improve comparability.

Influenza virus hospitalization rates among adults ≥65 years ranged from 180 to 304/100 000. Previous studies estimated influenza hospitalization incidence in France using the PMSI, with rates ranging from 36/100 000 (60–79 years) to 134/100 000 (80 years and over) over 2012–2017 [25]. Lemaitre *et al*. reported broader seasonal variability in influenza hospitalizations for respiratory causes in adults ≥65 years over 2010–2018, with annual rates ranging from 26 to 308/100,000, using a similar model approach [26].

Our study also provides one of the few estimates of RSV-related GP visits. Average annual incidence rates ranged from 2000 to 8000/100,000, higher in the 50–64-year age group than in the ≥65-year age group. RSV-attributable GP visit incidence was doubling that attributable to influenza. This higher rate of RSV in primary care should be considered when evaluating the burden. These observations do not take into account the higher vaccination rate for influenza among the older population.

Mortality rates increased with age ranging from 0.1 to 14/100,000, highlighting older adults’ vulnerability to severe disease from respiratory viruses such as RSV. These mortality rates were lower than the 37.9–44.3/100 000 observed by Haeberer *et al.* for adults aged ≥60 years [8], but similar to those reported by Hansen *et al.* for excess mortality in adults aged ≥65 years for RSV (14.7/100 000) and influenza (20.5/100 000) [4].

Comparing RSV severity to influenza severity, we observed much lower mortality for RSV than influenza mortality. Although RSV and influenza hospitalization rates were similar, RSV hospitalizations appeared more severe when analysing hospitalization characteristics. Patients with an RSV code were hospitalized for longer and were more likely to be admitted to intensive care, continuous care, or resuscitation units than those who did not have a diagnosis of RSV. These results may reflect the lack of routine testing for RSV in France, where routine testing is only performed in intensive care units, leading to the systematic capture of only the most severe RSV cases.

Both RSV and influenza virus can cause respiratory and cardiac events [27], and our incidence rates for GP visits and hospitalizations were ∼20% higher when we evaluated cardiorespiratory rather than respiratory symptoms alone. However, broad ICD-10 cardiac codes (I00–99) previously used to generate the time-series proved unreliable during modeling. For example, the estimated number of cardiorespiratory cases was lower than the number of respiratory cases. To increase model performance, we restricted our search to narrowly-defined cardiorespiratory conditions (identified using a limited set of diagnostic codes), which are known to be linked to RSV and influenza virus infections. Although these narrow definition cardiorespiratory codes were successfully used for the estimation of RSV-attributable GP visits and hospitalizations, we could not obtain adequate data to inform the mortality rate analysis. Nevertheless, these findings demonstrate how model accuracy can be improved by restricting the definition of diagnostic codes to reduce noise in the model.

Secondary diagnosis codes also merit consideration. While primary diagnosis codes reflect hospital admission reasons, secondary diagnosis codes are used to better characterize hospital stays. Our principal analysis used only primary codes, as secondary codes aren’t recorded for GP visits and death. However, using both primary and secondary codes for hospitalizations increased our estimates by 26–40%. A recent systematic literature review of ICD code validity studies found use of only primary ICD codes miss a number of pneumonia and lower respiratory tract infection events [28]. Analysis of hospital stays including an RSV ICD-10 code in any position revealed that most codes primary ICD-10 codes were related to respiratory and cardiac manifestations. This finding reinforces the value of including cardiac codes in the analysis. However, the top 10 primary codes outside the respiratory and cardiac categories were linked to pathologies unrelated to RSV infection (eg, oncology). Thus, considering both primary and secondary ICD-10 codes not only capture RSV infection leading to hospitalization but also RSV infection in hospitalized patients. Further research is needed to better understand the impact of RSV infection during hospitalization.

The estimated costs for respiratory hospitalizations attributable to RSV or influenza among patients aged ≥65 years were comparable (Supplementary Table 6). The mean total costs for cardiac hospitalizations attributable to RSV or influenza were 124 million € and 130 million € among patients aged >65 years, respectively, which aligns with the findings of recent influenza study [26].

Influenza virus infections displayed more seasonal variability than RSV, also observed in a U.S. study evaluating excess influenza and RSV mortality from 1999 to 2018 [4]. This likely reflects the circulation of distinct influenza virus subtypes (H1N1, H3N2, B), which varying severity by age and across season. H3N2 strains typically cause more severe disease in older adults, while B and H1N1 strains affect younger individuals [4, 29]. Other factors, like antigenic shift or drift, can create new strains capable of evading pre-existing immunity, while virus-vaccine mismatches can reduce vaccine effectiveness [30].

Model-based studies have inherent limitations. The use of different virus circulation indicators can significantly affect estimates. The estimates were modeled using bronchiolitis hospitalizations in children aged <2 years and influenza hospitalizations in adults aged ≥65 years. We retained all relevant codes from a study showing that RSV bronchiolitis was coded via J205, J210, and J219 [31]. We carried out sensitivity analyses (data not shown) using proxies from emergency room visits for bronchiolitis in children aged <2 years for RSV, and influenza-like illness for influenza from the sentinel GP network. Although influenza estimates remained unchanged, RSV data were significantly higher. Therefore, we adopted a conservative approach, relying on proxies derived solely from hospital data. We also investigated the impact of lags on the estimations (see Supplementary materials). Our model could not capture the burden of other seasonal virus like parainfluenza due to the unavailability of robust data, nor could it assess RSV related emergency visits. Further research on these two topics would be beneficial. In addition, further research should take into account short- and long-term post-pandemic changes in rates of infection such as observed by Falsey *et al.* [32].

## CONCLUSIONS

This study highlights the significant burden of RSV infection in adult’s ≥65 years in France. The incidence rate and economic burden of hospitalization were similar to the current burden of influenza. These underscore the need for model-based studies to better estimate and compare the burdens of respiratory diseases and generate real-world evidence to support prevention campaign implementation. Given the substantial clinical and economic burden of RSV infection, measures, such as vaccination campaigns, should be taken to prevent its spread.

## Supplementary Material

ofaf735_Supplementary_Data

## Supplementary Data

Supplementary materials are available at *Open Forum Infectious Diseases* online. Consisting of data provided by the authors to benefit the reader, the posted materials are not copyedited and are the sole responsibility of the authors, so questions or comments should be addressed to the corresponding author.
